# Overexpression of Secreted Frizzled-Related Protein 1 Inhibits Bone Formation and Attenuates Parathyroid Hormone Bone Anabolic Effects

**DOI:** 10.1359/jbmr.090719

**Published:** 2009-07-13

**Authors:** Wei Yao, Zhiqiang Cheng, Mohammad Shahnazari, Wewei Dai, Mark L Johnson, Nancy E Lane

**Affiliations:** 1 Department of Medicine, UC Davis Medical Center Sacramento, CA, USA; 2 Department of Oral Biology, UMKC School of Dentistry Kansas City, MO, USA

**Keywords:** sFRP1, transgenic, PTH, sex difference, bone mass

## Abstract

Secreted frizzled-related protein 1 (sFRP1) is an antagonist of Wnt signaling, an important pathway in maintaining bone homeostasis. In this study we evaluated the skeletal phenotype of mice overexpressing sFRP1 (*sFRP1 Tg*) and the interaction of parathyroid hormone (PTH) treatment and sFRP1 (over)expression. Bone mass and microarchitecture were measured by micro-computed tomography (µCT). Osteoblastic and osteoclastic cell maturation and function were assessed in primary bone marrow cell cultures. Bone turnover was assessed by biochemical markers and dynamic bone histomorphometry. Real-time PCR was used to monitor the expression of several genes that regulate osteoblast maturation and function in whole bone. We found that trabecular bone mass measurements in distal femurs and lumbar vertebral bodies were 22% and 51% lower in female and 9% and 33% lower in male *sFRP1 Tg* mice, respectively, compared with wild-type (WT) controls at 3 months of age. Genes associated with osteoblast maturation and function, serum bone formation markers, and surface based bone formation were significantly decreased in *sFRP1 Tg* mice of both sexes. Bone resorption was similar between *sFRP1 Tg* and WT females and was higher in *sFRP1 Tg* male mice. Treatment with hPTH(1-34) (40 µg/kg/d) for 2 weeks increased trabecular bone volume in WT mice (females: +30% to 50%; males: +35% to 150%) compared with *sFRP1 Tg* mice (females: +5%; males: +18% to 54%). Percentage increases in bone formation also were lower in PTH-treated *sFRP1 Tg* mice compared with PTH-treated WT mice. In conclusion, overexpression of sFRP1 inhibited bone formation as well as attenuated PTH anabolic action on bone. The gender differences in the bone phenotype of the *sFRP1 Tg* animal warrants further investigation. © 2010 American Society for Bone and Mineral Research

## Introduction

The wingless and Int1 (Wnt) family of secreted proteins regulates many aspects of cell growth, differentiation, and death during both embryogenesis and adult life.(1) The crucial role of Wnt signaling in mammalian bone homeostasis has been recognized in recent years.(2,3) Both canonical and noncanonical pathways are involved in coordinating proper bone development, formation, and growth during pre- and postnatal periods.(4,5) Canonical Wnt/β-catenin signaling supports osteogenic differentiation from mesenchymal stem cells (MSCs). During in vivo bone development, Wnt/β-catenin signaling prevents MSCs from differentiating into chondrocytes(6) and promotes osteoblast differentiation.(7) β-Catenin signaling in differentiated osteoblasts has been shown to negatively control osteoclast formation and bone resorption through an increase in osteoprotegerin production by osteoblasts.(8) Similar to other growth factors, Wnt signaling can be antagonized by secreted or intracellular inhibitors. Several known Wnt signaling inhibitors include secreted frizzled-related proteins (sFRPs), Wnt inhibitor factor I (WifI), the Dickkopf proteins (Dkks), and sclerostin (product of *Sost* gene). The sFRPs consist of at least seven members in vertebrates (sFRP1 to sFRP5, crescent, and sizzled) and contain a frizzled (Fz)–related cysteine-rich domain that can bind to both Wnt proteins and Fz but lack the intracellular sequence found in the Fz receptors. The sFRPs antagonize Wnt function by binding to the Wnt proteins and preventing Wnt/receptor activation. There is evidence to support the ability of sFRPs to inhibit both canonical and noncanonical Wnt signaling.(9) Overexpression of sFRP1 in human osteoblasts has been reported to accelerate the rate of osteoblast and osteocyte death.(10) Adult mice deficient in sFRP1 showed resistance to age-related bone loss.(11) The lack of sFRP1 in mice resulted in decreases in osteoblast and osteocyte apoptosis.(10) *sFRP1* knockout mice also have reduced bone anabolic responses to parathyroid hormone (PTH) treatment.(12) In addition to osteoblastogenesis, sFRP1 affects osteoclastogenesis because sFRP1 can directly bind RANKL(13) and/or inhibit the fusion of mononuclear cells,(14) thereby inhibiting osteoclast formation. Despite broad tissue distribution, ablation of *sFRP1* in mice did not affect blood and urine markers of bone resorption or organ function in most nonskeletal tissues.(11) Recently, a small molecule inhibitor (diarylsulfone sulfonamide) that can bind and inhibit sFRP1 was shown to stimulate bone formation,(15) suggesting that inhibition of sFRP1 may be a potential target to stimulate Wnt signaling to increase bone formation.

Since Wnt signaling is critical for osteoblast maturation, skeletal acquisition, and maintenance, we hypothesized that overexpression of sFRP1 in *sFRP1* transgenic mice (*sFRP1 Tg*) would reduce osteoblast number and activity such that these animals would have lower peak bone mass and reduced bone acquisition. Further, we were interested in the effect of PTH treatment in *sFRP1 Tg* mice.

## Materials and Methods

### Generation of the mice and experimental protocol

**Fig. 1:**
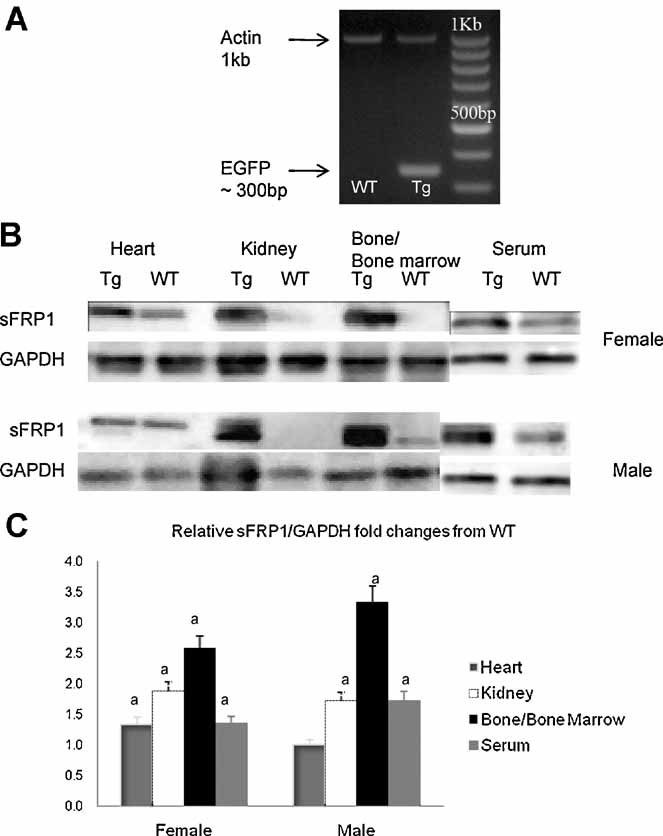
(*A*) Representative genotyping for *sFRP1*. The *sFRP1* transgenic mice (Tg) PCR product gives two bands at 1 kb (*Actin*) and 300 bp (*EGFP*), whereas wild-type (WT) mice have one band at 1 kb. (*B*) Proteins were extracted and analyzed by immunoblotting with antibodies against sFRP1 in selected tissues. *GAPDH* was used as a control. (*C*) Relative quantitative measurement of sFRP1 levels in selected tissues from both WT and *sFRP1 Tg* female and male mice. ^a^*p* < .05 from the WT mice.

The *sFRP1 Tg* mice used for this research project, STOCK Tg(sFRP1-EGFP)142Gsat/Mmcd and identification number 011017-UCD, were obtained from the Mutant Mouse Regional Resource Center (MMRRC), a National Center for Research Resource (NCRR)-National Institutes of Health (NIH)–funded strain repository and were donated to the MMRRC by the National Institute of Neurological Disorders and Stroke (NINDS)-funded Gene Expression Nervous System ATLas (GENSAT) engineering bacterial artificial chromosomes (BAC) transgenic project. The genotype has been modified to contain multiple copies of a modified BAC in which an *EGFP* reporter gene is inserted immediately upstream of the coding sequence of the *sFRP1* gene, producing site-specific expression of green fluorescent protein (GFP) in the gene of interest (*sFRP1*). EGFP expression is sFRP1-promoter driven. The genetic background of the *sFRP1 Tg* mouse was an FVB/N-Swiss Webster hybrid.(16) Mice genotypes were confirmed by a PCR protocol with a GFP R2 primer (TAGCGG CTGAAGCACTGC A). An actin primer was included in all PCR for genotyping. Electrophoresis was performed in 2% agarose gel at 90 V for 90 minutes. Wild-type (WT) mice had one band at 1000 bp for actin, whereas *sFRP1 Tg* mice had two bands at 1000 bp (actin) and at about 300 bp (sFRP1-EGFP fuse protein), respectively (Fig. 1*A*).

During the experiments, the mice were maintained on commercial rodent chow (Rodent Diet, Teklad, Madison, WI, USA) available *ad libitum* with 0.95% calcium and 0.67% phosphate. Mice were housed in a room that was maintained at 70 °F with a 12 hour light/dark cycle. All animals were treated according to the US Department of Agriculture (USDA) animal care guidelines with the approval of the UC Davis Committee on Animal Research.

At 12 weeks of age, both female and male WT or *sFRP1 Tg* mice were treated subcutaneously with either vehicle [phosphate-buffered daline (PBS)] (*n* = 10 to 11) or 40 µg/kg human PTH(1-34) (*n* = 12) (Bachem Bioscience, Inc., Torrance, CA, USA) 5×/week for 2 weeks. For all study animals, fluorochrome labeling with alizarin red (20 mg/kg s.c.) and calcein (10 mg/kg s.c.) were given 7 and 2 days before sacrifice to assess bone formation. Mice were killed by exsanguination from the heart at 14 weeks of age. Serum samples were obtained and were stored at −80°C prior to assessment of biochemical markers of bone turnover. The right femurs and fifth lumbar vertebral bodies were placed in 10% phosphate-buffered formalin for 24 hours and subsequently transferred to 70% ethanol for micro-computed tomography (µCT) and bone histomorphometry. The calvaria, lung, heart, liver, spleen, duodenum, kidney, left femurs, left tibiae, and ovaries or testes were collected for protein or RNA extractions for Western blot and real-time PCR analysis.

### µCT

The right distal femur from each of the animals was scanned using µCT (VivaCT 40, Scanco Medical, Bassersdorf, Switzerland) with an isotropic resolution of 10 µm in all three spatial dimensions. The scan was initiated at the lateral periosteal margin through the medial periosteal margin of the distal femur and 0.1 mm from the highest part of the growth plate continuing proximally for 2 mm. Mineralized bone was separated from the bone marrow with a matching-cube 3D segmentation algorithm. A normalized index, trabecular bone volume/total volume (BV/TV), was used to compare samples of varying sizes. Trabecular thickness (Tb.Th), trabecular separation (Tb.Sp), and trabecular number (Tb.N) also were calculated as we described previously.(17–19)

### Biochemical markers of bone turnover

Serum levels of the amino terminal propeptide of type 1 procollagen (P1NP), osteocalcin (OSC), osteoprotegerin (OPG), C-terminal telopeptides of type I collagen (CTX-I), and tartrate-resistant acid phosphatase 5b (TRAP5b) were measured using mouse sandwich ELISA kits from Biomedical Technologies (Stroughton, MA, USA) or Immunodiagnostic System (Fountain Hills, AZ, USA). The manufacturers' protocols were followed, and all samples were assayed in duplicate. A standard curve was generated from each kit, and the absolute concentrations were extrapolated from the standard curve. The coefficients of variation (CVs) for interassay and intraassay measurements were less than 10% for all assays and are similar to the manufacturers' references.(18,19)

### Bone histomorphometry

After fixation in 4% paraformaldehyde, the right distal femurs were dehydrated in graded concentrations of ethanol and xylene and embedded undecalcified in methyl methacrylate. The frontal sections (8 µm thick) were cut using a vertical-bed microtome (Leica/Jung 2255, Bannockbum, IL, USA) and affixed to slides coated with a 2% gelatin solution. Unstained 8 µm thick sections were used for assessing fluorochrome labeling and dynamic changes in bone. Bone histomorphometry was performed using semiautomatic image analysis (Bioquant Image Analysis Corporation, Nashville, TN, USA) linked to a microscope equipped with transmitted and fluorescent light. The analyses were performed in the secondary spongiosa of the distal femurs that included trabecular area between 100 and 300 µm distal to the growth plate and excluding the cortex. Bone turnover measurements included single- (sL.Pm) and double-labeled perimeter (dL.Pm), interlabel width (Ir.L.Wi), and osteoclast surface. These indices were used to calculate mineralizing surface (MS/BS) and mineral apposition rate (MAR), bone formation rate (BFR/BS), and percentage of osteoclast surface (Oc.S).(17–19)

### RNA preparation and RT-PCR

Tibiae were dissected free of the articular cartilage and soft tissue and then flash frozen in liquid nitrogen. Total RNA from long bone and bone marrow were isolated using a modified two-step purification protocol employing homogenization (PRO250 Homogenizer, 10 mm × 105 mm generator, PRO Scientific IN, Oxford, CT, USA) in Trizol (Invitrogen, Carlsbad, CA, USA) followed by purification over a Qiagen RNeasy column (Qiagen, GmbH, Hilden, Germany). Real-time PCR was carried out on an ABI Prism 7300 (Applied Bioscience, Foster City, CA, USA) in a 25 µL reaction consisted of 12.5 µL 2× SYBER Green Mix (SABioscience, Inc., Frederick, MD, USA), 0.2 µL cDNA, 1 µL primer pair mix, and 11.3 µL H_2_O. Primer sets for real-time PCR were purchased from Superarray (Frederick, MD, USA). All the test genes were expressed relative to a control gene, β-actin or *GAPDH*, using the delta-delta *Ct* method. The results were expressed as fold changes from WT group, where fold change is 2^−ΔΔ*Ct*^.

### Primary bone marrow stromal cell culture

Bone marrow cells were harvested from 14-week-old WT or *sFRP1 Tg* mice of both sexes. After removing the soft tissue, the bone marrow from the left femurs was flushed out several times in a solution of PBS, antibiotics (penicillin-streptomycin, 20% by volume), and Fungizone (5% by volume) under sterile conditions. The cells were plated in primary medium at 10 × 10^6^ in 60 mm dishes or 3 × 10^6^ in six-well plates in primary medium with α-MEM culture medium, 10% fetal bovine serum, 1% antibiotics (penicillin-streptomycin), and 0.1% Fungizone. The primary medium was removed on day 5, leaving only the adherent cells on the plate. Then the cells were replenished with the secondary medium containing all the ingredients of the primary medium plus 50 µg/mL ascorbic acid and 10mM β-glycerophosphate. Cell proliferation was measured on day 5 using the BrdU Immunofluorescence Assay Kit from Roche Applied Science (Indianapolis, IN, USA). On day 14 of the culture, alkaline phosphatase (ALP) levels were measured by incubating the cells with *p*-nitrophenyl phosphate substrate (Sigma-Aldrich, St. Louis, MO, USA) at room temperature for 30 minutes. The optical absorbance was read at 405 nm. After the absorbance was taken, protein concentrations from these wells were measured with the Bio-Rad DC protein assay kit (Bio Rad, Hercules, CA, USA) using bovine serum albumin as standard. The ALP activities were normalized to protein content. ALP value was expressed as amount of ALP that hydrolyzes 1 µmol *p*-nitrophenol/mg protein per minute. Another set of cells was fixed and stained for ALP. On day 21 of the cell culture, a set of cells was fixed in 10% neutral buffered formalin and stained with alizarin red (AR) to monitor mineralization nodule formation.

### Western blots/antibodies

Protein lysates from various mouse tissues were prepared by homogenizing 10 to 20 mg of tissue from WT or *sFRP1 Tg* mice in cold RadioImmuno Precipitation Assay (RIPA) lysis buffer. Debris was removed by centrifugation, and protein concentration was measured by the BioRad DC kit (Bio Rad). Immunoprecipitates were analyzed by SDS-PAGE and Western blot analyses using standard conditions and the following antibodies: rabbit polyclonal anti-sFRP1 (Abcam, 1:230), mouse monoclonal anti-active dephosphorylated β-catenin (Upstate, 1:200), rabbit polyclonal anti-GAPDH (1:200), and normal mouse or rabbit IgG (Santa Cruz Biotechnology, Santa Cruz, CA, USA). Immune complexes were visualized by incubation with horseradish peroxidase–conjugated secondary antibody. After washing, protein bands were detected with a chemiluminescence (ECL) detection system (Amersham Biosciences, Piscataway, NJ, USA). Quantification of the intensity of the bands in the autoradiograms was performed using a Kodak imaging system and analyzed by SCION IMAGE (Frederick, MD, USA). Normalization was performed with GAPDH antibody.

### Statistics

The group means, standard deviations, and percent differences between the *sFRP1 Tg* and the WT animals were calculated for all the outcome variables. The effects of genotype (WT and *sFRP1 Tg*), treatment (vehicle and PTH), sex (male and female), and their interactions were analyzed using a multiple analysis of variance (MANOVA, SPSS 12, SPSS, Chicago, IL, USA). Differences were considered significant at *p* < .05.

## Results

Body weights and hindlimb lengths were similar between WT and *sFRP1 Tg* mice for both females and males. RT-PCR revealed increased *sFRP1* gene expression in lung, liver, spleen, kidney, ovary/testis, and bone in both female and male *sFRP1 Tg* mice (range 1.2- to 5-fold) compared with WT controls. Western blotting for sFRP1 in these various tissues (i.e., heart, kidney, bone/bone marrow, serum) confirmed the PCR changes (see Fig. 1*B*, *C*).

### Mice with overexpression of sFRP1 develop osteopenia and have lower bone formation in both sexes

**Fig. 2:**
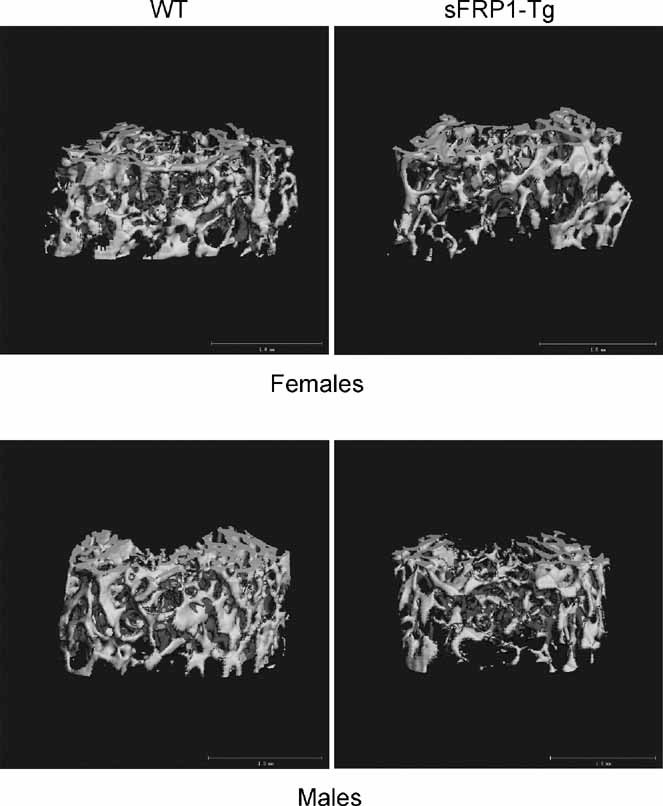
Representative µCT images of the distal femoral trabecular bone from WT and *sFRP1 Tg* mice at the age of 14 weeks. Scale bars: 1.0 mm.

µCT analysis was performed on the distal femoral metaphyses (DFMs) and the fifth lumbar vertebral bodies (LVB5s) from 14-week-old mice. The trabecular bone mass (BV/TV) in the DFMs and LVB5s was 22% and 9% lower in the female *sFRP1 Tg* mice. In the male *sFRP1 Tg* mice, BV/TV was 51% and 33% lower in the DFMs and LVB5s, respectively, compared with the WT mice. Bone lose was associated with reduced trabecular thickness (Tb.Th) in the *sFRP1 Tg* mice. Midshaft femoral cortical bone thickness was 13% lower in female and 21% lower in male *sFRP1 Tg* mice compared with the WT mice (Tables 1 and 2 and Fig. 2).

**Table 1:** Trabecular Architectural Changes in the Distal Femoral Metaphyses

Groups	BV/TV (%)	Tb.N (#/mm)	Tb.Th (*µ*m)	Ct.Th (*µ*m)
Females				
WT + Vehicle (n = 12)	25.9 ± 1.6	5.1 ± 0.2	56.6 ± 1.8	160 ± 5
Tg + Vehicle (n = 11)	20.2 ± 2.7	4.7 ± 0.4	54.1 ± 2.0	139 ± 15
WT + PTH (n = 9)	39.6 ± 7.1	6.3 ± 0.9	71.0 ± 11.1	170 ± 4
Tg + PTH (n = 10)	21.3 ± 6.1	5.3 ± 1.2	61.1 ± 18.6	143 ± 16
Males				
WT + Vehicle (n = 11)	24.8 ± 9.8	5.0 ± 0.3	52.8 ± 9.0	164 ± 12
Tg + Vehicle (n = 12)	12.1 ± 2.3	4.7 ± 0.6	41.4 ± 2.7	129 ± 14
WT + PTH (n = 11)	62.3 ± 13.7	6.2 ± 0.8	116.6 ± 8.6	254 ± 23
Tg + PTH (n = 10)	14.3 ± 3.8	4.9 ± 0.6	47.4 ± 4.7	143 ± 8
MANOVA				
Genotype	0.01	NS	0.03	0.003
Sex	NS	NS	NS	0.005
Genotype * Sex	0.03	NS	0.003	NS
PTH	0.01	NS	0.002	0.002
PTH*Genotype	NS	NS	NS	NS
PTH*Sex	0.04	NS	0.004	0.004
PTH*Genotype*Sex	NS	NS	NS	NS

Tg, sFRP1-trangenic; BV/TV: bone volume; Tb.N: trabecular number; Tb.Th: trabecular thickness; Ct.Th, cortical bone thickness. NS, non-significant. Data is presented as Mean ± standard deviation.

**Table 2:** Trabecular Architectural Changes in the 5^th^ Lumbar Vertebral Bodies

Groups	BV/TV (%)	Tb.N (#/mm)	Tb.Th (*µ*m)
Females			
WT + Vehicle (n = 12)	22.6 ± 2.8	4.7 ± 0.9	49.1 ± 2.6
Tg + Vehicle (n = 11)	20.5 ± 3.2	4.7 ± 0.5	48.6 ± 1.9
WT + PTH (n = 9)	29.3 ± 8.0	5.3 ± 1.0	55.2 ± 5.6
Tg + PTH (n = 10)	21.1 ± 1.1	4.7 ± 1.2	47.4 ± 8.0
Males			
WT + Vehicle (n = 11)	21.1 ± 2.2	4.9 ± 0.1	46.8 ± 2.7
Tg + Vehicle (n = 12)	14.1 ± 3.7	4.7 ± 0.3	40.3 ± 4.1
WT + PTH (n = 11)	49.4 ± 16.6	5.3 ± 0.4	128.9 ± 10.2
Tg + PTH (n = 10)	21.7 ± 5.4	5.1 ± 0.8	44.3 ± 3.7
MANOVA			
Genotype	0.04	NS	NS
Sex	NS	NS	NS
Genotype*Sex	0.02	NS	0.01
PTH	0.01	NS	0.04
PTH*Genotype	NS	NS	NS
PTH*Sex	NS	NS	0.03
PTH*Genotype*Sex	NS	NS	NS

Tg, sFRP1-trangenic; BV/TV: bone volume; Tb.N: trabecular number; Tb.Th: trabecular thickness. NS, non-significant. Data is presented as Mean ± standard deviation.

P1NP and OPG, indicators for osteoblast activity, did not differ between the *sFRP1 Tg* and WT mice in females. Osteocalcin, a bone marker associated with osteoblast maturation, decreased by 33% in female *sFRP1 Tg* mice compared with the WT female mice (*p* < .05). However, in male mice, P1NP, osteoclacin, and OPG decreased by 63%, 12%, and 20%, respectively, in the *sFRP1 Tg* mice compared with the WT mice (*p* < .05) (Table 3). Surface-based bone formation (BFR/BS), measured by histomorphometry, decreased by 68% and 48% in female and male *sFRP1 Tg* mice respectively, compared with their sex-matched WT controls (see Table 3).

**Table 3:** Bone Formation Parameters

Groups	P1NP (ng/mL)	Osteocalcin (ng/mL)	OPG (ng/mL)	BFR/BS (µm/d)
Females				
WT + Vehicle (n = 12)	7.5 ± 1.7	21.6 ± 3.4	2.12 ± 0.7	0.37 ± 0.1
Tg + Vehicle (n = 11)	8.1 ± 1.6	14.4 ± 2.4	1.98 ± 0.05	0.12 ± 0.1
WT + PTH (n = 9)	26.8 ± 4.6	30.3 ± 6.8	2.31 ± 0.5	0.44 ± 0.1
Tg + PTH (n = 10)	13.2 ± 3.0	21.2 ± 7.0	2.06 ± 0.5	0.27 ± 0.1
Males				
WT + Vehicle (n = 11)	25.1 ± 1.4	11.8 ± 1.5	1.95 ± 0.2	0.55 ± 0.1
Tg + Vehicle (n = 12)	9.2 ± 1.3	10.3 ± 1.7	1.55 ± 0.3	0.29 ± 0.3
WT + PTH (n = 11)	62.4 ± 10.3	21.8 ± 5.6	2.82 ± 0.6	1.04 ± 0.2
Tg + PTH (n = 10)	30.3 ± 13.2	19.8 ± 5.7	2.49 ± 0.9	0.34 ± 0.1
MANOVA				
Genotype	0.01	0.05	0.05	0.001
Sex	0.0001	0.05	NS	0.01
Genotype*Sex	0.03	NS	0.02	0.02
PTH	0.0001	0.03	0.01	0.001
PTH*Genotype	0.03	NS	NS	0.05
PTH*Sex	0.01	NS	NS	0.02
PTH*Genotype*Sex	0.05	NS	NS	0.01

BFR/BS: Bone formation rate/bone surface; NS, non-significant. Data is presented as Mean ± standard deviation.

### Overexpression of sFRP1-inhibited osteoblast differentiation and function

**Fig. 3:**
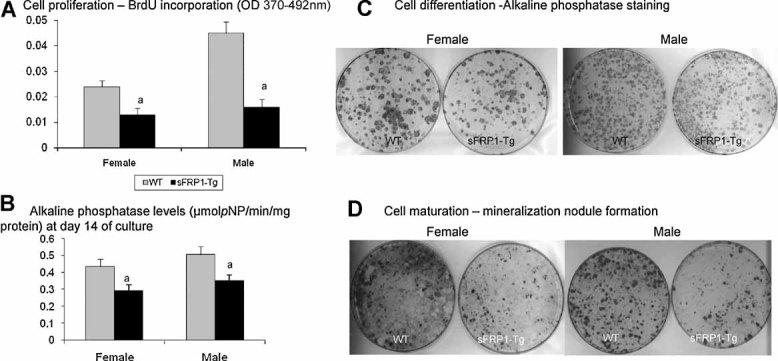
Bone stromal cell culture from 14-week-old WT or *sFRP1 Tg* mice. (*A*) Cell proliferation in WT or *sFRP1 Tg* mice. Bone marrow cells derived from either WT or *sFRP1 Tg* mice were cultured on a 96-well plate for 4 days, and then the cells were incubated with BrdU for 2 hours. Absorbance at 370 to 392 nm was measured with a microplate reader. (*B–D*) Bone marrow cells were extracted and cultured with ascorbic acid and β-glycerophosphate for 14 days. After ALP levels were obtained (*B*), the cells were stained with ALP staining to monitor ALP-positive colony formation (*C*). Alizarin red staining (AR) was performed at day 21 to monitor mineralization nodule formation (*D*).

In order to evaluate the effects of systemic sFRP1 overexpression on osteoblastogenesis in vitro, we collected bone marrow cells and cultured the cells in osteogenic medium. Cell proliferation was measured by BrdU incorporation on day 5 of the culture, and osteogenic differentiation was determined by measuring both ALP and mineralization nodule formation with AR staining. Cell proliferation was 46% lower in female and 64% lower in male *sFRP1 Tg*–derived cells compared with WT-derived cell cultures (Fig. 3*A*). On day 14 of the cell culture, in cells derived from *sFRP1 Tg* mice, ALP value was about 30% lower in both females and the males compared with WT male mice (see Fig. 3*B*), and the cells had less ALP-positive colony formation (see Fig. 3*C*). Cells derived from *sFRP1 Tg* mice had lower mineralization nodule formation, which was about 50% lower in the females and males compared with WT male mice (see Fig. 3*D*).

**Fig. 4:**
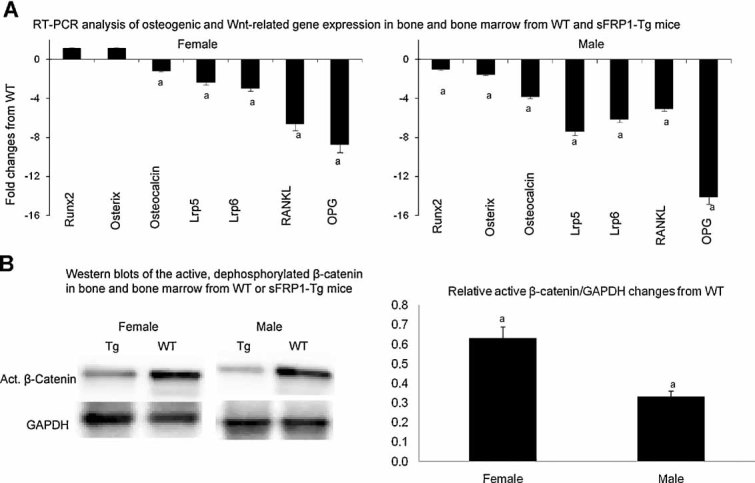
Expression of several genes that regulate osteoblast maturation and function in whole bone from 14-week-old WT or *sFRP1 Tg* mice. (*A*) RNA was extracted from bone and bone marrow of the tibiae and real-time PCR was performed to monitor gene expression that was associated with osteoblast differentiation and maturation (*Runx2*, *Osterix*, *Osteocalcin*, *RANKL*, and *OPG*) or Wnt signaling (*Lrp5* and *Lrp6*). (*B*) Protein was extracted from bone and bone marrow from WT or *sFRP1 Tg* mice and subjected to Western blotting of the active dephosphorylated β-catenin. ^a^*p* < .05 from the WT.

We also evaluated osteoblast differentiation and maturation in vivo. We extracted RNA from the whole bone (tibiae) and performed real-time PCR on selected gene markers for osteoblast differentiation and maturation (*Runx2*, *Osterix*, and *Osteocalcin*), key regulators in Wnt signaling (*Lrp5* and *Lrp6*), and the bone turnover cytokines (*RANKL* and *OPG*). Osteocalcin expression was decreased in the female *sFRP1 Tg* mice, whereas all three osteoblastic maturation gene markers were decreased one- to threefold in the male *sFRP1 Tg* mice compared with the WT controls (Fig. 4*A*). The expression levels of Lrp5 and Lrp6, coreceptors for Wnt/β-catenin signaling, were significantly lower (about threefold) in female and more than sixfold lower in male *sFRP1 Tg* mice compared with WT mice (see Fig. 4*A*). The expression levels of RANKL and OPG were 6- to 14-fold lower in the *sFRP1 Tg* mice compared with WT mice (see Fig. 4*A*). “Active” (nonphosphorylated) β-catenin expression was about 40% lower in bone/bone marrow in female *sFRP1 Tg* mice and was about 60% lower in male *sFRP1 Tg* mice compared with WT mice (see Fig. 4*B*).

### Overexpression of sFRP1 did not affect bone resorption in vivo but induced higher osteoclastogenesis in vitro compared with the WT mice

The sFRP1 overexpression increased TRAP5b, CTX-1, and osteoclast surface (Oc.S) by 56%, 34% and 38%, respectively, in female *sFRP1 Tg* mice compared with WT controls. In male *sFRP1 Tg* mice, sFRP1 overexpression increased TRAP5b, CTX-1, and osteoclast surface by 48%, 39% and 37%, respectively, compared with WT controls (Table 4).

**Table 4:** Bone Resorption Parameters

Groups	TRAP5b (U/L)	CTX-1(U/L)	Oc.S (%)
Females			
WT + Vehicle (n = 12)	3.9 ± 0.8	9.6 ± 2.0	3.7 ± 0.7
Tg + Vehicle (n = 11)	6.1 ± 0.9	12.9 ± 2.1	5.1 ± 0.3
WT + PTH (n = 9)	5.3 ± 1.7	11.9 ± 1.1	4.1 ± 0.1
Tg + PTH (n = 10)	5.6 ± 1.2	13.0 ± 3.3	5.7 ± 0.1
Males			
WT + Vehicle (n = 11)	3.1 ± 1.3	8.1 ± 0.8	4.5 ± 0.6
Tg + Vehicle (n = 12)	4.6 ± 0.9	11.3 ± 2.1	6.8 ± 0.9
WT + PTH (n = 11)	4.9 ± 0.8	10.1 ± 2.1	5.8 ± 0.1
Tg + PTH (n = 10)	5.0 ± 1.8	14.2 ± 1.8	7.9 ± 0.4
MANOVA			
Genotype	0.01	0.05	0.001
Sex	NS	NS	NS
Genotype*Sex	NS	NS	0.05
PTH	NS	NS	NS
PTH*Genotype	NS	0.03	0.04
PTH*Sex	NS	NS	0.01
PTH*Genotype*Sex	NS	NS	0.001

Tg, sFRP1-trangenic; Oc.S, osteoclast surface. NS, non-significant. Data is presented as Mean ± standard deviation.

### Overexpression of sFRP1 attenuated the increase in bone mass and bone formation following PTH treatment that was observed in the WT controls

Since sFRP1 is associated with a reduction in osteoblast maturation and function, we hypothesized that *sFRP1 Tg* mice may have a reduced anabolic response to PTH treatment compared with WT controls. In female WT mice treated with PTH for 14 days, BV/TV of the DFMs and LVB5s was 53% and 30% higher than in the vehicle-treated WT group (see Table 1). In male WT mice treated with PTH, BV/TV of the DFMs and LVB5s was 151% and 134% higher than in male vehicle-treated WT mice (see Table 1).

In female PTH-treated WT mice, bone-formation parameters, including serum P1NP, osteocalcin, OPG, and BFR/BS, were increased by 257%, 40%, 9%, and 19%, respectively, compared with WT mice. In female PTH-treated *sFRP1 Tg* mice, these parameters were increased by 63%, 47%, 4% and 125%, respectively, compared with the vehicle-treated *sFRP1 Tg* mice (see Table 3). In male WT mice treated with PTH, the percentage increases in PINP, osteocalcin, OPG, and BFR/BS following PTH treatment were 148%, 85%, 44% and 89% compared with the male vehicle-treated WT mice. PTH treatment in *sFRP1 Tg* male mice increased these parameters by 229%, 92%, 60% and 17%, respectively, compared with vehicle-treated *sFRP1 Tg* mice (see Table 3).

Osteogenic marker gene expression in female and male WT mice treated with PTH were increased (range 1.6- to 2-fold) compared with vehicle-treated WT mice. PTH treatment in WT mice decreased *RANKL* expression by about 1.5-fold, whereas it increased *OPG* expression by 2-fold. However, the gene expression patterns in *sFRP1 Tg* female and male mice treated with PTH were similar to those of vehicletreated *sFRP1 Tg* mice (see Fig. 4*A*). PTH treatment increased active β-catenin concentration in bone and bone marrow by more than twofold in WT mice but failed to do so in *sFRP1 Tg* mice (see Fig. 4*B*).

**Fig. 5 fig05:**
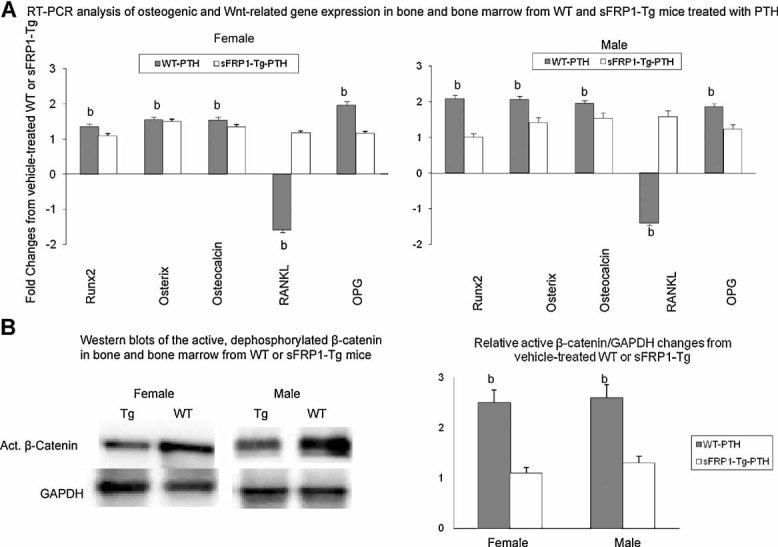
Expression of several genes that regulate osteoblast maturation and function in whole bone from 12-week-old WT or *sFRP1 Tg* mice treated with PTH for 2 weeks. (*A*) RNA was extracted from bone and bone marrow from the tibiae of WT or *sFRP1 Tg* mice treated with PTH. Real-time PCR was performed to monitor gene expression that was associated with osteoblast differentiation and maturation (*Runx2*, *Osterix*, *Osteocalcin*, *RANKL*, and *OPG*) or Wnt signaling (*Lrp5* and *Lrp6*). (*B*) Protein was extract from bone and bone marrow of WT or *sFRP1 Tg* mice treated with PTH and subjected to Western blotting of the active dephosphorylated β-catenin. ^b^*p* < 0.05 from vehicle-treated WT or *sFRP1 Tg* mice.

After determining that sFRP1 overexpression impaired PTH-induced bone formation, we then analyzed its effect on bone resorption. PTH tended to increase these bone-resorption parameters in WT mice by 10% to 50%, but these changes did not reach significant levels. In the *sFRP1 Tg* mice, PTH treatment did not change these bone-resorption parameters compared with vehicle treatment. However, PTH treatment further increased TRAP5b, CTX-1, and Oc.S in male *sFRP1 Tg* mice by 9%, 25%, and 16%, respectively, compared with the vehicle-treated male *sFRP1 Tg* mice (see Table 4).

## Discussion

This study determined that overexpression of sFRP1 in mice results in an osteopenic phenotype characterized by an inhibition of bone formation, with males being more severely affected than females. In addition, sFRP1 overexpression attenuated PTH initial anabolic action on bone, as characterized by substantially less gain in bone mass and lower osteoblast maturation gene activations in both sexes. PTH further increased osteoclast surface and bone resorption (CTX-1) in the male *sFRP1 Tg* mice, suggesting that increased bone resorption also may play a role in the reduced bone anabolic effects observed in male transgenic mice.

### Effects of overexpression of sFRP1 in bone

The phenotype changes we have described for *sFRP1 Tg* mice are different from those reported for the *sFRP1* knockout mouse.(10,11) The *sFRP1* knockout (KO) mice and WT mice had similar trabecular bone mass at 3 months of age, and age-related bone loss was observed in the WT mice but not in the *sFRP1* KO mice. However, at 14 weeks of age, *sFRP1 Tg* mice had a significantly lower amount of trabecular and cortical bone mass, with the male *sFRP1 Tg* mice being more severely osteoporotic than the female mice. The *sFRP1 Tg* mice had lower parameters of bone formation measured by both serum bone-formation markers and surface-based bone histomorphometry compared with WT mice. Lower bone formation accounts for most of bone loss seen in these *sFRP1 Tg* mice.

The *sFRP1 Tg* mice had broad tissue distribution increases in sFRP1 expression. Lung, liver, kidney, ovary/testis, and bone had 1.6- to 4-fold higher sFRP1 levels than the WT mice. The β-catenin levels in bone and bone marrow were lower in the *sFRP1 Tg* mice, especially the males. Our study found that systemic overexpression of sFRP1 lowered Wnt/β-catenin signaling in bone and inhibited bone marrow cell proliferation and osteogenic differentiation that resulted in lower osteoblast maturation and function. Although our transgenic model was not specific to osteoblasts, our findings suggest that systemic sFRP1 overexpression appeared to affect osteogenic differentiation and bone formation. Recently, Bodine and colleagues reported that a small molecule inhibitor (diarylsulfone sulfonamide) that bound to human sFRP1 inhibited canonical Wnt signaling measured by TCF-luciferase reporter assay in vitro.(15) Although an in vivo evaluation of bone effects is not yet available for this sFRP1 inhibitor, it is possible that selective inhibition of sFRP1 may be a potential target to stimulate Wnt signaling that increases bone formation.

Wnt/β-catenin signaling regulates both mesenchymal stem cells' commitment to osteoblast differentiation and osteoclast maturation and activity through the OPG/RANKL system.(20–22) Secreted FRP1 can directly bind RANKL(13) and/or inhibit the fusion of mononuclear cells,(14) thereby inhibiting osteoclast formation. We found that in vivo bone resorption was higher in the *sFRP1 Tg* mice. Our findings that sFRP1 overexpression increased bone resorption in the male mice were similar to the findings of Diarra and colleagues,(23) who reported the effects of Dkk-1, another Wnt antagonist, on bone resorption. Dkk-1 overexpression stopped osteoblast maturation at the preosteoblast stage. As a result, more RANKL and less OPG were produced, resulting in increased or accelerated osteoclastogenesis.(23) Secreted FRP1 may bind to the soluble Wnt agonists and prevent Wnt signaling through the frizzled/Lrp5/Lrp6, which would arrest osteoblast proliferation and differentiation. The decrease in mature osteoblasts might enhance bone resorption by lowering the production of OPG, increasing the RANKL/OPG ratio, and permitting RANKL-induced osteoclastogenesis.(24)

Interestingly, sexual dimorphism in Wnt/β-catenin signaling of bone formation and bone mass often exists. In humans, there is a stronger association between Lrp5 polymorphism and bone mass in males compared with females.(25–27) In females, two common variants of Lrp5 were reported to be associated with vertebral fracture risk.(28) Sexual dimorphism in Wnt/β-catenin signaling also has been reported in mice that have been genetically modified for inhibition of Wnt/ β-catenin signaling. In mice null for *Lrp5*, lumbar vertebral trabecular bone mass was 10% lower in females and 43% lower in males compared with their aged-matched WT counterparts, with lumbar compression strength lowered by nearly 30% in females and by 50% in males.(29) On the other hand, in mice that overexpressed LRP5 G171V, trabecular bone mass was twofold higher in both the female and male transgenic mice at 3 months of age.(30–32) However, when the mice aged, the trabecular bone mass was about 100% higher in female and 162% higher in male transgenic mice compared with WT mice.(31) Adult female *sFRP1* KO mice had 179% more trabecular bone mass and male *sFRP1* KO mice had 142% more trabecular bone mass than WT controls by 35 weeks of age.(11) Additional experiments are now under way to investigate the sexual differences of Wnt/β-catenin signaling in bone.

### Effects of sFRP1 overexpression on bone response to PTH

PTH is a known anabolic agent for bone because it increases bone osteoblast maturation and osteoblast activity and prolongs osteoblast lifespan.(33–36) PTH anabolic effects are partially mediated though the Wnt signaling pathway. PTH treatment directly increased the levels of β-catenin and the transcriptional activity of TCF/LEF in osteoblastic cell lines.(37,38) PTH also may achieve its anabolic effects partially by the suppression of *Sost*(19,39–41) and *Dkk1*(19,37) transcription. Interestingly, mice null for Lrp5, a coreceptor for Wnt, did not have an increase in bone mass with PTH treatment.(29,42) However, another coreceptor for Wnt, Lrp6, was reported to form a receptor complex with type 1 parathyroid hormone receptor (PTH1R) and increase osteogenesis. This Lrp6/PTH1R signaling pathway may help to explain PTH's anabolic action on bone.(43) When we treated *sFRP1 Tg* and WT mice with PTH for a relatively short period (14 days), we found that sFRP1 overexpression inhibited the initial bone anabolic response to intermittent PTH treatment. Interestingly, in *sFRP1* KO mice, PTH also had less anabolic effects on bone.(12) Our results and studies from *sFRP1* KO mice suggest that PTH downregulation of sFRP1 is required for stimulation of the Wnt signaling needed for osteoblastogeneis. In the KO mice, bone mass is already high owing to an increased Wnt signaling; consequently, the additional effects of PTH on Wnt signaling result in only a minor anabolic effect. In overexpressing mice, Wnt signaling is low, and and without the suppressive effects of the hormone on sFRP1, full PTH stimulation of Wnt signaling is delayed.

Once osteoblasts begin to differentiate into the preosteoblasts, they synthesize RANKL, which is the most potent activator of osteoclast maturation and activity. Mature osteoblasts, defined by gene expression of osterix and ALP, do not synthesize RANKL and instead synthesize OPG, an inhibitor of RANKL that belongs to the tumor necrosis factor (TNF) receptor family.(44,45) We found that sFRP1 overexpression lowered OPG mRNA expression to a larger degree than it lowered RANKL expression, suggesting that sFRP1 overexpression inhibits osteoblast maturation more than its inhibition on osteoblast differentiation. Less OPG production in return potentiated bone resorption.

In our transgenic model, sFRP1 levels were higher in the tissues (bone and bone marrow), as well as in the circulation (sFRP1 in serum). A targeted expression of sFRP1 to cells of the osteoblast lineage will need to be examined to study the direct effects of sFRP1 on osteoblast. One question that remains unanswered is whether sFRP1 works at the level of bone/bone cells or through some other tissue site, as suggested for the role of Lrp5 in regulation of bone mass.(46) While we have no evidence to oppose such a possibility in the case of sFRP1 overexpression, the fact that sFRP1 works by binding to Wnt and thereby decreasing intracellular β-catenin levels, rather than targeting one of the Lrp5/Lrp6 coreceptors, would seem to favor a more direct mechanism acting on the bone compartment.

This study has a number of strengths, including the in vivo and in vitro assessments of sFRP1 overexpression on bone metabolism. However, there are also a number of shortcomings. We evaluated only 12- to 14-week-old animals in this study and were not able to report age-related changes following sFRP1 overexpression. Our PTH treatment period was relatively short and only allowed us to study the mechanistic aspects of the interaction. We observed a delay in the response of bone mass to PTH treatment in the *sFRP1 Tg* mice. However, since increases in bone formation parameters were similar between the genotypes following PTH treatment, it is likely that longer PTH treatment or higher dose of PTH may overcome the inhibition of sFRP1 overexpression on osteogenesis and bone formation. In addition, we did not evaluate the effects of sFRP1 antibody or the addition of Wnt protein in the cultures. Evaluating these effects may enable us to better understand the mechanisms of the sFRP1's impact on osteoblastogenesis.

In summary, systemic overexpression of sFRP1 inhibited the osteoblast maturation and function in bone. The sFRP1 overexpression also attenuated the PTH rapid effects on bone augmentation. The sex difference in the skeletal phenotypes of this *sFRP1 Tg* mouse strain is unexpected and warrants further investigation.
